# Three questions for identifying chemically intolerant individuals in clinical and epidemiological populations: The Brief Environmental Exposure and Sensitivity Inventory (BREESI)

**DOI:** 10.1371/journal.pone.0238296

**Published:** 2020-09-16

**Authors:** Raymond F. Palmer, Carlos R. Jaén, Roger B. Perales, Rodolfo Rincon, Jacqueline N. Forster, Claudia S. Miller

**Affiliations:** Department of Family and Community Medicine, University of Texas Health Science Center at San Antonio, San Antonio, Texas, United States of America; Chinese Academy of Medical Sciences and Peking Union Medical College, CHINA

## Abstract

The Quick Environmental Exposure and Sensitivity Inventory (QEESI) is a validated questionnaire used worldwide to assess intolerances to chemicals, foods, and drugs, and has emerged as the gold standard for assessing chemical intolerance (CI). Despite a reported prevalence of 8–33%, epidemiological studies and routine primary care clinics rarely assess CI. To help address this gap, we developed the Brief Environmental Exposure and Sensitivity Inventory (BREESI)—a 3-item CI screening tool. We tested the BREESI’s potential to predict whether an individual is likely to be classified as chemically intolerant if administered the 50-item QEESI. We recruited 293 participants from a university-based primary care clinic and through online participation. The statistical sensitivity, specificity, and positive and negative predictive values of the BREESI were calculated against the validated QEESI. Ninety percent (90%) of participants answering “yes” to all three items on the BREESI fit the QEESI criteria for being *very suggestive* of CI based upon their chemical intolerance and symptom scores (positive predictive value = 90%). For participants endorsing two items, 93% were classified as either *very suggestive* (39%) *or suggestive* (54%) of CI (positive predictive value = 87%). Of those endorsing only one item, 13% were classified as *very suggestive* of CI, and 70% as *suggestive*. Of those answering “No” to all of the BREESI items, 95% were classified as *not suggestive* of CI (i.e., negative predictive value = 95%). The BREESI is a versatile screening tool for assessing potential CI useful for clinical and epidemiological applications, based upon individuals’ past adverse responses in a variety of settings. Just as health care professionals routinely inquire about latex allergy to prevent adverse reactions, the BREESI provides an essential screen for CI. Together, the BREESI and QEESI provide new diagnostic tools that may help predict and prevent future adverse reactions to chemicals, foods, and drugs.

## Introduction

There is growing international concern over intolerances to chemicals [1, 2], foods [3, 4], and drugs [5]. Up to one-quarter of the U.S. population reports being either “especially” or “unusually” sensitive to certain chemicals, with about 5% reporting physician-diagnosed CI [6]. Population-based surveys estimate prevalence between 8% and 33% in several countries [6–8]. In a university family practice clinic, Katerndahl et al. reported that 20% of patients were chemically intolerant [9]. At least 1 in 10 US adults have well-documented food allergies, and 1 in 5 report food intolerances [10]. Among US children, the reported prevalence of food allergy is 38.7%, with peanut, milk, and shellfish among the top offenders [11]. A large US electronic medical records study showed that 2.1% of health plan patients reported three or more drug intolerances [12]. Similarly, a UK survey of more than 25,000 inpatients with a documented drug intolerance, showed that 4.9% had Multiple Drug Intolerance Syndrome (MDIS), defined as 3 or more adverse reactions to drugs—suggesting cross intolerances [13].

The specific mechanism(s) underlying CI have been elusive, but there is evidence for a general disease process called “Toxicant-Induced Loss of Tolerance (TILT)”, which parsimoniously captures the wide variety of symptoms and intolerances to chemicals, foods, and medicines reported by researchers among individuals with this condition [14–16].

TILT is a two-stage disease mechanism initiated by a major exposure event or a series of exposures (Stage I, Initiation). Affected individuals experience multi-system symptoms triggered by everyday chemicals, foods, and medications that never bothered them before and do not bother most people (Stage II, Triggering). Initiating exposures include chemical spills, pesticides, cleaning agents, solvents, combustion products, drugs and medical devices, molds, and indoor air contaminants associated with construction or remodeling [14–17].

Despite a relatively high population prevalence rate, CI often goes undiagnosed. Part of the challenge physicians face is that current assessment tools are too time-consuming for routine use. To address this need, and the need for a useful epidemiological tool, we developed and tested the following three-item questionnaire, the Brief Environmental Exposure and Sensitivity Inventory (BREESI) (Box 1):

**Box 1. The Brief Environmental Exposure and Sensitivity Inventory (BREESI).**

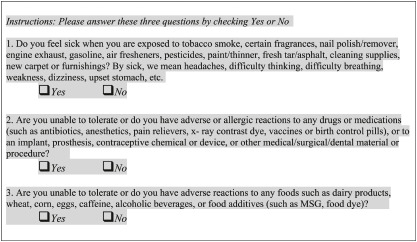

The items on the BREESI help gauge an individual’s tendency to react adversely to diverse substances representing three major exposure categories (chemicals, foods, and drugs) covered by the Quick Environmental Exposure and Sensitivity Inventory (QEESI). The 50-item QEESI is a validated, self-administrable questionnaire geared toward differentiating individuals with CI from the general population [17–19]. Researchers in over a dozen countries have used the QEESI and it has become the reference standard for measuring CI [20–23].

*QEESI Scores*: The QEESI has 4 scales: Symptom Severity, Chemical Intolerances, Other Intolerances, and Life Impact. The scales contain 10 items, each rated from 0 to 10:

0 = “not a problem” to 10 = “severe or disabling problem.” Scale totals range from 0–100. There is also a 10-item Masking Index which gauges ongoing exposures (such as caffeine, alcohol, or tobacco use) that can affect individuals’ awareness of their intolerances as well as the intensity of their responses to environmental exposures [18, 19].

There are three classifications for CI, based on the QEESI Chemical Intolerance and Symptom scales. Scores greater than or equal to 40 on each scale are *very suggestive* of CI. Scores from 20–39 on one or both scales are *suggestive* of CI. Scores less than 20 on both scales are *not suggestive* of CI [18, 19].

The BREESI’s three questions pertaining to chemical, food, and drug intolerances were derived from the validated QEESI. Although the QEESI chemical exposure scale is designed to assess adverse reactions to ten specific chemical exposures, the BREESI compresses this scale into a single “yes” or “no” question. Similarly, the BREESI compresses items on the QEESI Other Intolerances Scale that pertain to food and drug intolerances into one “yes” or “no” question for foods, and another for drugs.

Our goal was to create a brief but sensitive instrument for assessing CI in clinical settings and epidemiological research. Here, we investigate the sensitivity and specificity of the BREESI as well as its positive and negative predictive values for a clinical population referenced to the QEESI.

## Materials and methods

### Study sample

Trained research staff randomly approached 749 individuals in the waiting room of the University of Texas Health System’s Family and Community Medicine Clinic, 180 of whom agreed to complete both the BREESI and QEESI. Participants had to be at least 18 years of age with no other inclusion criteria. An additional 113 respondents completed the same surveys online through an email link to online versions of the QEESI and BREESI. Informed consent was obtained in the clinic or digitally online. This project was one component of our overall environmental health research program whose purpose is to improve health outcomes of CI individuals by helping them identify and eliminate environmental triggers in their homes. This study was approved by the University of Texas Health Science Center San Antonio Internal Review Board (approval number HSC20150821H).

### Statistical analysis

A logistic regression model was used to obtain the Odds-Ratios with 95% confidence intervals and the c-statistic for the BREESI as a predictor of CI. Potential confounding variables were included in a multivariate model. All analyses were conducted using SAS statistical software [24].

To determine the predictive value of the BREESI, we calculated the positive and negative predictive values of the three BREESI items against the established QEESI ranges. Sensitivity and specificity of the BREESI were also calculated using a receiver operator curve (ROC).

## Results

Of the 293 participants, 207 (75.3%) were female. There were no gender differences between online and clinic participants (p = .56). On average, online participants were somewhat older (53 versus 47 years, (p < .002). Compared to clinic participants, a significantly greater percentage of online participants fit the QEESI criteria for being *very suggestive* of CI (47.0% versus 25.4% respectively, p < .006). There were no significant differences in the number of BREESI items endorsed by online and clinic respondents (1.53 vs 1.40, respectively, p = .31).

Table 1 summarizes the demographics by QEESI CI category. Values with different superscripts in the table signify statistically different values between groups. On average, those whose scores were *Very Suggestive* or *Suggestive* of CI were older than the *Not Suggestive* group (p < .01). There were also significantly more females in the *Very Suggestive* or *Suggestive* CI groups compared to the other two groups (p < .001). Shown in Table 1 are the means of the QEESI Chemical and Symptom Scale scores we used to classify participants into CI categories. Also shown are their Other Intolerance Scale scores. As anticipated, these scores are all highly significantly different from one another across the three CI groups (P < .0001), i.e., *Very Suggestive*, *Suggestive*, and *Not Suggestive*.

**Table 1:** Characteristics of study sample (N = 293) by group by QEESI category.

	Very Suggestive of CI (N = 98)	Suggestive of CI (N = 105)	Not Suggestive of CI (N = 90)
**% Female**	87.8%^a^	73.6%^b^	63.3%^b^
	n = 86 females, 12 males	n = 64 females, 23 males (n = 18 missing information)	n = 57 females, 33 males
**Age**			
mean (SD)	54.7 (10.6)^a^	50.7 (13.1)^a^	41.1 (15.3)^b^
**QEESI Scale Scores**,			
mean (SD)			
*Chemical Intolerance*	73.1 (16.2)^a^	32.1(18.9)^b^	5.1 (4.8)^c^
*Symptom Severity*	68.4 (15.2)^a^	30.0(15.8)^b^	4.4 (5.1)^c^
*Other Intolerances*	50.9 (20.1)^a^	16.1 (12.7)^b^	4.5 (7.4)^c^

Different superscript letters next to the data values indicate significant differences. Values with the same letter superscript indicate no statistical difference.

**Fig 1:**
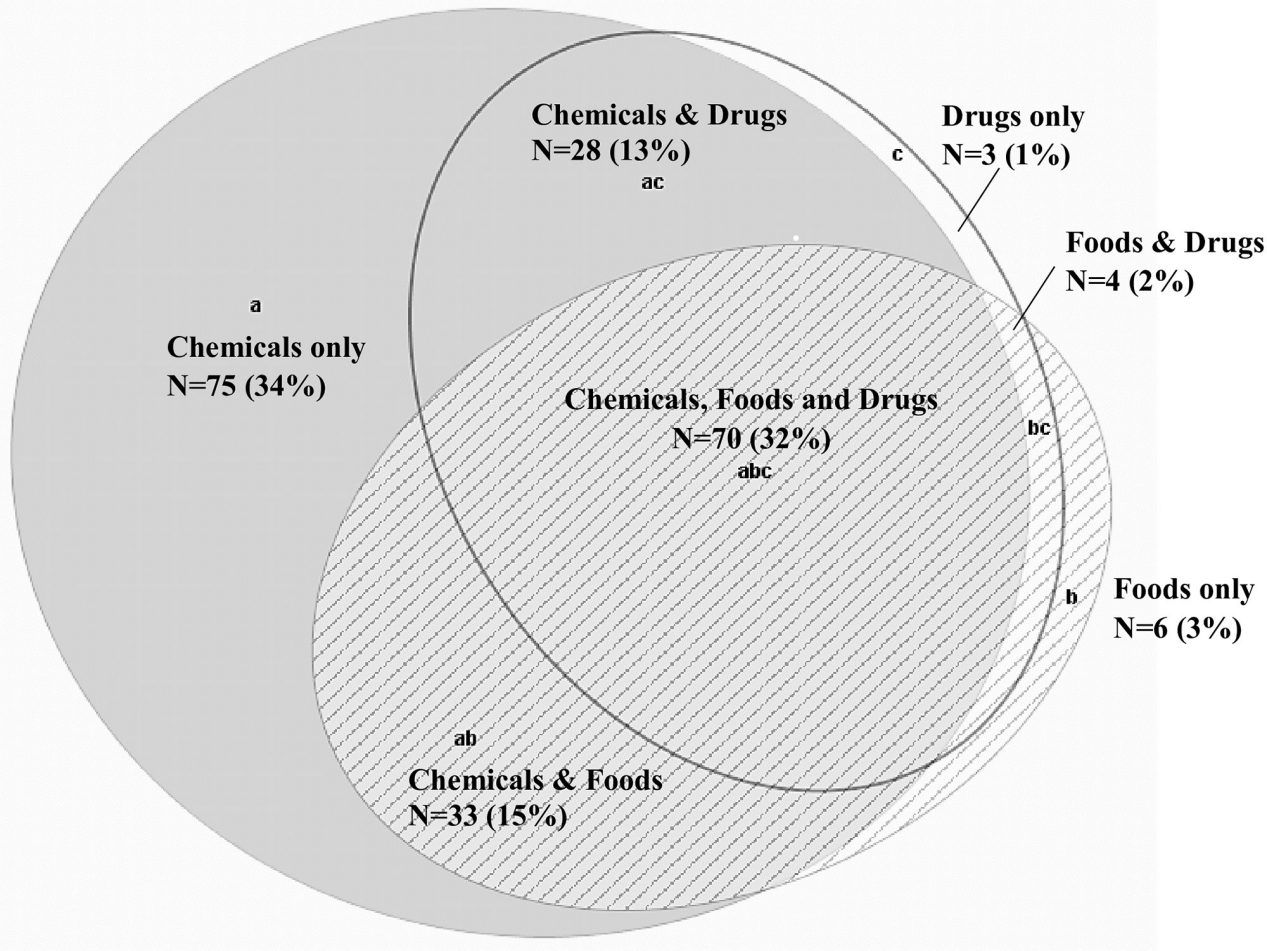
Overlap between those choosing one or more BREESI items (N = 219/293, 75%).

Response rates for the total sample on individual BREESI items were as follows: 25.3% (n = 74) of the 293 participants did not endorse any item; 28.7% (n = 84) chose only one item; 22.2% (n = 65) chose two items; 23.8% (n = 70) chose all three BREESI items. The Venn diagram in Fig 1 shows the number and percentage of the different response combinations on the BREESI. Whether one, two, or three BREESI items were chosen, overall, the chemical item was endorsed most often.

**Fig 2:**
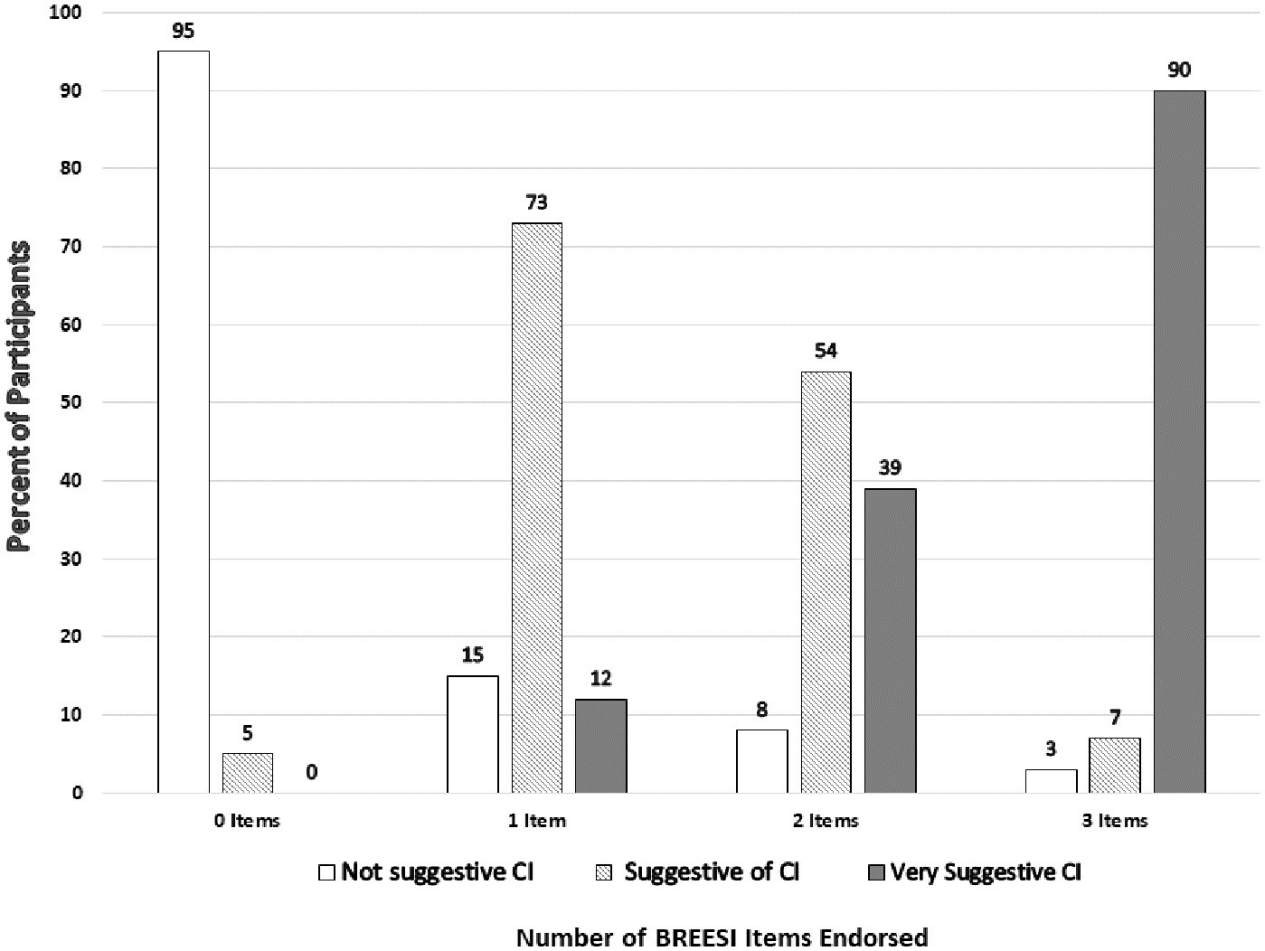
Number of items endorsed on the BREESI versus QEESI Chemical Intolerance category (N = 293).

As shown in Fig 2, 90% of participants answering “yes” to all three BREESI items fit the QEESI category “*very suggestive* of CI”, i.e., QEESI Chemical Intolerance and Symptom scale scores both ≥ 40. Ninety-seven (97%) of the participants who answering “yes” to all three items on the BREESI fit the QEESI category of being “*very suggestive* or *suggestive* of CI”. This gives the BREESI a positive predictive value of 97%.

Of those who endorsed two BREESI items, 93% fit the QEESI categories of being either “*very suggestive”* (39%) *or “suggestive”* (54%) of CI. This yields a positive predictive value of 92% for the BREESI when two items are chosen.

Among those who endorsed only one BREESI item, 13% fit the QEESI category of “*very suggestive* of CI”. Notably, in this clinical population, even among those who chose only one of the three BREESI items, 73% fit the QEESI category “*suggestive* of CI” (positive predictive value = 84%).

Of those who did not endorse any of the BREESI items, 95% fit the QEESI category “*not suggestive* of CI”. Thus, the BREESI had a negative predictive value of 95%. This means that the BREESI correctly identified those with no chemical intolerance 95% of the times when no items were chosen.

A logistic regression model was used to assess the odds of being *very suggestive* of CI based on the responses to the BREESI. With only the BREESI as a predictor, there was a 12-fold increase in the probability of CI with each additional item chosen on the BREESI (Odds Ratio = 12.0, 95% Confidence Interval = 6.6–21.6, p < .0001). The concordance statistic (c) was 0.95 (95% CI = 90.3–96.9), calculated using the method of Delong et al. [25]. The (c) statistic specifies the ability of the BREESI to discriminate CI respondents from non-CI. This result indicates that 95% of the time, BREESI scores correctly classified individuals’ QEESI status as *very suggestive* of CI. This is reinforced by the ROC curve of 0.95 (95% CI = 0.92–0.97), with sensitivity = 90.0 and specificity = 87.0.

The results of the logistic model, adjusted for the covariates of age, gender, and online vs clinic participation, yielded an Odds Ratio = 10.6, 95% (Confidence Interval = 5.8–19.4, p < .0001). Of these covariates, only age was statistically significant (Odds Ratio = 1.04, 95% CI = 1.01–1.08, p < .03).

## Discussion

We have developed and tested a screening questionnaire for chemical, food, and drug intolerances that can be administered in less than one minute. If indicated by the BREESI score, the QEESI can be administered in 15–20 minutes. Previously, tallying symptoms and symptom severities, as well as taking detailed exposure histories, could take hours, deterring some clinicians from evaluating patients for CI.

The BREESI exhibits excellent positive and negative predictive validity, as well as sensitivity and specificity, when evaluated against the QEESI reference standard. These results suggest that false positive outcomes are minimized and imply a high probability of CI when there is a positive BREESI screening [26]. We therefore propose the BREESI as an efficient tool for determining potential CI in a range of health care settings and for epidemiological studies. Further replication of the BREESI’s performance in other populations is needed to confirm its validly.

The BREESI is not a substitute for the QEESI, but rather is a time-saving tool to identify individuals with CI in medical clinics or epidemiological studies. Identifying those who are, or are not, likely to have CI using this sensitive screening tool greatly reduces clinical assessment time. Specifically, the BREESI may be useful for researchers, clinicians, health plans, as well as workplace or community investigations. We strongly recommend that an individual who endorses any one of the three BREESI items take the full QEESI to help identify specific chemical, food, and drug triggers. By using a cutoff of one BREESI item, health care workers and researchers are unlikely to overlook anyone who may be affected. Individuals who endorse 2 or 3 BREESI items may be at greater risk of developing additional intolerances or adverse reactions to exposures or medications.

In the past, there has not been a widely agreed-upon case definition for the diverse symptoms and conditions related to CI. For decades, this lack of consensus has thwarted research on CI, just as the lack of consensus regarding a case definition for “Gulf War Illness” impeded research [27]. Clinicians today have difficulty diagnosing TILT-related conditions because health care givers receive little or no training regarding these intolerances, which do not involve classical immunoglobins (e.g. IgE, IgG).

Practitioners who could benefit from using the BREESI include most primary care doctors, allergists, dermatologists, occupational medicine doctors, dentists, psychologists, and psychiatrists. These clinicians commonly see patients with multi-system health complaints, cognitive confusion, fatigue, and depression. Using the BREESI and QEESI together could help identify patients who are more chemically intolerant and help them avoid or minimize their exposures. This is not unlike health care professionals routinely inquiring about latex allergy in order to prevent adverse reactions. Clinicians and epidemiologists who work with chemically exposed individuals or communities need to be conversant with the two-stage TILT disease mechanism if they hope to make sense of the seemingly unrelated health problems and many symptom triggers these individuals report.

“Too many symptoms in too many organ systems” frustrates clinicians and patients alike [28]. It is all too easy to dismiss TILT as being stress-induced or psychosomatic. Stress can and does occur simultaneously. Stress can be one consequence of TILT or any other toxic exposure. However, the initiating and triggering exposures must also be addressed. It is important to recognize that the usual medications prescribed for stress, such as antidepressants or anxiolytics, often exacerbate symptoms of TILT.

New-onset (or marked worsening) of chemical, food and drug intolerances are hallmarks of TILT, much as fever signifies possible infection. The BREESI can help physicians and researchers differentiate between stress and potential chemical, food, and drug intolerances. We propose administering the BREESI to patients at every visit and is as important as asking them about prior adverse reactions to latex or antibiotics.

Exposures that may initiate TILT in susceptible individuals include indoor air contaminants such as mold and volatile organic compounds, oil spills, chemical releases, fracking, or burn pits, as well as exposure events such as the EPA’s sick building episode and the World Trade Center disaster. In addition to major events, everyday exposures to pesticides, fragranced personal care or other fragranced products can initiate or trigger TILT in susceptible individuals. Interestingly, in our clinical experience, whether 1, 2 or 3 BREESI items were chosen, the chemical item is the most endorsed BREESI item (Fig 1). This is consistent with our and others’ experience with the QEESI. The early research conducted in the development of the QEESI demonstrates that acute or chronic, low dose exposures to a wide range of chemicals including pesticides; off gassing associated with new building construction or remodeling; Gulf war exposures; and implanted medical devices are associated with the *initiation* of CI. Subsequently, affected individuals report allergic-like responses to structurally unrelated substances that include foods and drugs [18, 19, 29]. This observation is consistent with the worldwide CI literature [1, 30–32].

We found a statistically higher percentage of women in the “*Very suggestive of CI*” QEESI category (Table 1). This likely was not due to recruitment bias, which was random. The preponderance of women with CI is consistent with global population-based studies [32–35]. In a Japanese population-based study, CI among females was approximately twofold higher than in males [7].

QEESI scores can also be assessed at intervals in order to follow symptoms over time or document responses to treatments or exposure avoidance [30]. Participating individuals should always have the option of discussing results with investigators or their personal physicians.

## Conclusion

Twenty percent of primary care patients report chemical, food, and/or drug intolerances [9]. Many who might be helped go undetected. The BREESI provides a rapid means of identifying intolerances, thereby providing clinicians and epidemiologists with a useful, new screening tool. Individuals who answer “yes” to any of the three BREESI items should also complete the QEESI, which can help patients, physicians, and public health practitioners identify salient chemical, dietary, and drug triggers, gauge the life impact of intolerances, and assess the efficacy of the interventions (whether exposure avoidance, medications, or nonpharmacological methods). Healthcare professionals routinely ask about a history of adverse reactions to latex or antibiotics. Similarly, we urge practitioners, medical practices, and health plans to adopt the BREESI in order to screen their patients for CI. If the BREESI screen is positive, then patients should complete a QEESI and give copies to each of their health care providers.

## Supporting information

S1 Data Minimal BREESI data set. (XLSX)

## References

[1] Hojo S, Mizukoshi A, Azuma K, Okumura J, Ishikawa S, Miyata M, et al Survey on changes in subjective symptoms, onset/trigger factors, allergic diseases, and chemical exposures in the past decade of Japanese patients with multiple chemical sensitivity. International Journal of Hygiene and Environmental Health. 2018; 221:8: 1085–1096. 30115513 10.1016/j.ijheh.2018.08.001

[2] Steinemann A. 2018 National Prevalence and Effects of Multiple Chemical Sensitivities. Journal of Occupational and Environmental Medicine 60(3):e152–e156 10.1097/JOM.0000000000001272 29329146 PMC5865484

[3] Young E, Stoneham MD, Petruckevitch A, Barton J, Rona R. A population study of food intolerance. The Lancet. 1994:343(8906):1127–1130, 10.1016/S0140-6736(94)90234-8. 7910231

[4] Rona R, Keil T, Summers Colin, Gislason David, Zuidmeer Laurian, Sodergren Eva, et al The prevalence of food allergy: A meta-analysis. Journal of Allergy and Clinical Immunology. Volume 120, Issue 3, 2007, Pages 638–646, 10.1016/j.jaci.2007.05.026. 17628647

[5] Macy E. Chapter 16—Multiple Drug Intolerance Syndrome, Editor(s): David A. Khan, Aleena Banerji. Drug Allergy Testing. Elsevier, 2018: 165–168, ISBN 9780323485517, 10.1016/B978-0-323-48551-7.00016-X.

[6] Caress SM, Steinemann AC. Prevalence of multiple chemical sensitivities: a population-based study in the southeastern United States. Am J Public Health. 2004; 94(5): 746–747. 10.2105/ajph.94.5.746 15117694 PMC1448331

[7] Azuma et al, 2015 Prevalence and Characteristics of Chemical Intolerance: A Japanese Population-Based Study. Arch Environ Occup Health; 70:341–353. 10.1080/19338244.2014.926855 25137616

[8] Kreutzer R, Neutra RR, Lashuay N. Prevalence of people reporting sensitivities to chemicals in a population-based survey. Am J Epidemiol. 1999; 150(1): 1–12. 10.1093/oxfordjournals.aje.a009908 10400546

[9] Katerndahl DA, Bell IR, Palmer RF, Miller CS. Chemical intolerance in primary care settings: prevalence, comorbidity, and outcomes. Ann Fam Med. 2012; 10(4): 357–365. 10.1370/afm.1346 22778124 PMC3392295

[10] Gupta RS, Warren CM, Smith BM, et al Prevalence and Severity of Food Allergies Among US Adults. JAMA Netw Open. Published online January 04, 20192(1):e185630 10.1001/jamanetworkopen.2018.5630 PMC6324316 30646188

[11] Gupta RS, Springston EE, Warrier MR, Smith B, Kumar R, Pongracic J, et al The Prevalence, Severity, and Distribution of Childhood Food Allergy in the United States. Pediatrics. 7 2011, 128 (1) e9–e17; 10.1542/peds.2011-0204 21690110

[12] Macy E, Ho NJ. Multiple drug intolerance syndrome: prevalence, clinical characteristics, and management. Ann Allergy Asthma Immunol. 2012;108(2):88–93. 10.1016/j.anai.2011.11.006 22289726

[13] Omer HM, Hodson J, Thomas SK.,& Coleman JJ. (2014). Multiple drug intolerance syndrome: a large-scale retrospective study. Drug safety,37(12), 1037–1045. 10.1007/s40264-014-0236-x 25362509 PMC4243008

[14] Miller CS. The compelling anomaly of chemical intolerance. Ann NY Acad Sci. 2001; 933:1–23. 10.1111/j.1749-6632.2001.tb05810.x 12000012

[15] Miller CS. Toxicant-induced loss of tolerance—an emerging theory of disease? Environ Health Perspect. 1997; 105 Suppl 2:445–453. 9167978 10.1289/ehp.97105s2445 PMC1469811

[16] Genuis SJ. Sensitivity-related illness: The escalating pandemic of allergy, food intolerance and chemical sensitivity. Science of The Total Environment: 2010 408 (24): 6047–6061. 10.1016/j.scitotenv.2010.08.047 20920818

[17] Miller, CS. Toxicant-Induced Loss of Tolerance-The QEESI©. Townsend Letter for Doctors and Patients. 2001:85–89.

[18] Miller CS, Prihoda TJ. The Environmental Exposure and Sensitivity Inventory (EESI): a standardized approach for measuring chemical intolerances for research and clinical applications. Toxicol Ind Health. 1999a; 15(3–4):370–385. 10416289 10.1177/074823379901500311

[19] Miller CS, Prihoda TJ. A controlled comparison of symptoms and chemical intolerances reported by Gulf War veterans, implant recipients and persons with multiple chemical sensitivity. Toxicol Ind Health. 1999b;15(3–4):386–397. 10.1177/074823379901500312 10416290

[20] Hojo S, Kumano H, Yoshino H, Kakuta K, & Ishikawa S. Application of Quick Environment Exposure Sensitivity Inventory (QEESI^©^) for Japanese population: study of reliability and validity of the questionnaire. Toxicol Ind Health. 2003; 19(2–6):41–49. 10.1191/0748233703th180oa 15697173

[21] Jeon BH, Lee SH, & Kim HA. A validation of the Korean version of QEESI^©^ (The Quick Environmental Exposure and Sensitivity Inventory). Korean J Occup Environ Med. 2012; 24(1):96–114.

[22] Nordin S & Andersson L. Evaluation of a Swedish version of the quick environmental exposure and sensitivity inventory. Int. Arch. Occup. Envi. 2010;83(1):95–104. 10.1007/s00420-009-0427-4 19468745

[23] Skovbjerg S, Berg ND, Elberling J & Christensen KB. Evaluation of the quick environmental exposure and sensitivity inventory in a Danish population. J Environ Public Health. 2012:304314 Epub 2012 Jan 12. 10.1155/2012/304314 22529872 PMC3317206

[24] SAS Institute Inc. 2014 SAS^®^ 9.4 Statements: Reference, Third Edition Cary, NC: SAS Institute Inc.

[25] DeLong ER, DeLong DM, Clarke-Pearson DL. Comparing the areas under two or more correlated receiver operating characteristic curves: a nonparametric approach. Biometrics. 1988; 44:837–845. 3203132

[26] Trevethan R. Sensitivity, Specificity, and Predictive Values: Foundations, Pliabilities, and Pitfalls in Research and Practice Frontiers in Public Health 2017 (5):307, 10.3389/fpubh.2017.00307 29209603 PMC5701930

[27] Nettleman M. Gulf War Illness: Challenges Persist. Trans Am Clin Climatol Assoc. 2015; 126: 237–47. 26330683 PMC4530672

[28] Ashford NA, Miller CS. Chemical Exposures: Low Levels and High Stakes, 2nd Edition. January 1998. Wiley, NJ. 464 pages. ISBN: 978-0-471-29240-1.

[29] Miller CS, and Mitzel HC. Chemical Sensitivity Attributed to Pesticide Exposure Versus Remodeling. Archives of Environmental Health. 1997; 50(2): 119–129. 10.1080/00039896.1995.9940889 7786048

[30] Yun M-J, Kang D-M, Lee K-H, Kim Y-K, Kim J-E. Multiple chemical sensitivity caused by exposure to ignition coal fumes: a case report. Annals of Occupational and Environmental Medicine. 2013; 25:32 10.1186/2052-4374-25-32 24472417 PMC3923363

[31] Hojo S, Sakabe K, Ishikawa S, Miyata M, Kumano H. Evaluation of subjective symptoms of Japanese patients with multiple chemical sensitivity using QEESI©. Environmental Health and Preventive Medicine. 2009 (14): 267–275 (2009). 10.1007/s12199-009-0095-8 PMC2728252 19603254

[32] Azuma K, Jinno H, Tanaka-Kagawa T, Sakai S. Risk assessment concepts and approaches for indoor air chemicals in Japan, International Journal of Hygiene and Environmental Health, 2020 (225). 10.1016/j.ijheh.2020.113470. 32050149

[33] Dantoft MT, Andersson L, Nordin S, Skovbjerg S. Current Rheumatology Reviews. 2015 (11),2: 167–184. 10.2174/157339711102150702111101 26088215

[34] Bell IR, Baldwin CM. Multiple Chemical Sensitivity Women and Health (Second Edition); 2013: 1379–1394. Academic Press.

[35] Lago BE, Puiguriguer FJ, Rodriguez EM. Multiple chemical sensitivity: Clinical evaluation of the severity and psychopathological profile. Med Clin (Barc) 2016; 146(3):108–11. 26654553 10.1016/j.medcli.2015.09.016

